# Socioeconomic Status and Later-Life Health: Longitudinal Evidence From Europe and China

**DOI:** 10.1093/geroni/igab046.1864

**Published:** 2021-12-17

**Authors:** Mengling Cheng, Nicolas Sommet, Daniela Jopp, Dario Spini

**Affiliations:** 1 University of Lausanne, Lausanne, Vaud, Switzerland; 2 University of Lausanne Faculty of Social and Political Sciences, Lausanne, Vaud, Switzerland

## Abstract

Scholars are divided as to how the protective effect of SES on health (the SES-health gradient) varies over the later-life course: The age-as-leveler perspective suggests that the SES-health gradient weakens with age, whereas the cumulative (dis)advantages perspective suggests that it strengthens with age. To clarify this, we used SHARE 2004-2017 (73,407 respondents from 19 European countries) and CHARLS 2011-2018 (8,370 Chinese respondents). Congruent with the age-as-leveler perspective, growth curve models revealed that the overall protective effect of SES on multimorbidity was weaker for older than younger adults (the country-specific effects were significant in two thirds of the case). We interpret this as a selection effect. However, the within-participant protective effect of SES on multimorbidity did not vary over the later-life course (the country-specific effects were nonsignificant in the majority of the case). Findings suggest that extant cross-sectional studies should be interpreted with caution and that longitudinal, cross-national studies are needed.

